# Supplementary material for: The role of intra-guild indirect interactions in assembling plant-pollinator networks
Source: Nat Commun. 2023 Sep 18;14:5797. doi: 10.1038/s41467-023-41508-y (PMC10507117; doi:10.1038/s41467-023-41508-y)
Supplement: Supplementary file 1 — Supplementary Information [file 41467_2023_41508_MOESM1_ESM.pdf]

Supplementary Information for

The role of intra-guild indirect interactions in assembling plant-pollinator networks

Sabine Dritz*[1], Rebecca A. Nelson[1], Fernanda S. Valdovinos*[1]

[1]Department of Environmental Science and Policy, University of California Davis, 350 East Quad, Davis, CA 945616 United States

*Corresponding authors are Sabine Dritz sjdritz@ucdavis.edu and Fernanda S. Valdovinos fvaldovinos@ucdavis.edu

Supplementary Information, in the order that it is first mentioned in the main text, includes:

Table S1: Parameter values used for the assembly models.

Table S2: Motif frequency among attempted and established colonizers for each guild and assembly model.

Table S3: Motif frequency after establishment, extinctions, and the subsequent colonization event for each guild and assembly model.

Figure S1: Simulated and empirical network structures and degree distributions.

Figure S2: Motif frequency among attempted and established early, middle, and late stage colonizers for each guild and assembly model.

Table S1: Parameter values used for the assembly models.

Species' parameters were drawn randomly from uniform distributions with the mean and variances listed below. To ensure species turnover in our assembly model, we modified the mean and variance of key parameters from values used in previous studies indicated by bolded values.

Definition	Symbol	Dimension	Mean | Variance
State Variables			
Density of plant population i	P_i	individuals area^{-1}	variable
Density of animal population j	A_j	individuals area^{-1}	variable
Total density of floral resources of plant population i	R_i	mass area^{-1}	variable
Foraging effort of j on i	α_{ij}	None	variable
Parameters			
Visitation efficiency	τ_j	visits area time^{-1} individuals^{-1} individuals^{-1}	1 | **0.5**
Expected number of seeds produced by a pollination event	e_{ij}	individuals visits^{-1}	0.8 | 0.1
Per capita mortality rate of plants	μ_i^P	time^{-1}	0.001 | 0.1
Conversion efficiency of floral resources to pollinator births	c_{ij}	individuals mass^{-1}	0.2 | 0
Per capita mortality rate of pollinators	μ_j^A	time^{-1}	0.001 | 0
Pollinator extraction efficiency of resource in each visit	b_{ij}	individuals visits^{-1}	0.4 | 0
Maximum fraction of total seeds that recruit to plants	g_i	None	0.4 | 0.1
Inter-specific competition coefficient of plants	u_i	area individuals^{-1}	**0.02 | 1**
Intra-specific competition coefficient of plants	w_i	area individuals^{-1}	**0.04 | 0**
Production rate of floral resources	β_i	mass individuals^{-1} time^{-1}	**0.8 | 1**
Attachability of pollen to pollinator's body	ϵ_i	None	**4 | 1**
Self-limitation parameter of resource production	ϕ_i	time^{-1}	0.04 | 0.1
Adaptation rate of foraging efforts of pollinators	G_j	None	2 | 0

Table S2: Motif frequency among attempted and established colonizers for each guild and assembly model. Here we consider colonizer's motif groups at the moment they arrive to the network before undergoing transformations due to extinctions or subsequent establishments. This table includes data from 121 simulations (see Methods) of network assembly for each assembly model. Each simulation constructs a network from 50 colonization events of three plants and three pollinators. However, the first 9 colonizers of each guild are excluded from our motif analysis because the network is not large enough to analyze. Additionally, species who do not have indirect interactions (i.e., pollinators or plants interacting directly with only specialist plants or pollinators, respectively) were excluded. This includes species who have indirect partners at the moment of their introduction but lose them over the following 4,000 timesteps (the timeframe of our motif analysis). Given these exclusions, we observed over 16,000 attempted colonizers of each guild for each assembly model.

Species guild	Assembly model	Motif group	Motif frequency attempted	Motif frequency after establishment	Establishment rate
Plant	W/ AF	Spec-Spec	743	60	0.08
		Spec-Gen	7227	1722	0.24
		Gen-Spec	2926	10	0.003
		Gen-Gen	5490	130	0.02
		Total	16386	1922	
	W/O AF	Spec-Spec	713	187	0.26
		Spec-Gen	7293	609	0.08
		Gen-Spec	2460	1339	0.54
		Gen-Gen	5911	1510	0.26
		Total	16377	3645	
Pollinator	W/ AF	Spec-Spec	2760	809	0.29
		Spec-Gen	5254	1059	0.20
		Gen-Spec	5415	2189	0.40
		Gen-Gen	2960	1034	0.35
		Total	16389	5091	
	W/O AF	Spec-Spec	1251	488	0.39
		Spec-Gen	6761	1689	0.25
		Gen-Spec	3087	1142	0.37
		Gen-Gen	5277	1596	0.30
		Total	16376	4915	

Table S3: Motif frequency after establishment, extinctions, and the subsequent colonization event for each guild and assembly model. Colonizer's motif groups can transform due to intra-guild indirect specialist extinctions and subsequent establishments (Fig. 8). If all indirect specialists are excluded, motif groups "Spec-Spec" and "Gen-Spec" will be transformed into "Spec-Gen" and "Gen-Gen", respectively (Fig. 8A, B). Indirect specialists cannot be excluded from motif groups "Spec-Gen" and "Gen-Gen" because there are none present, for that reason those cells are greyed out. If indirect specialists establish during the subsequent colonization event, "Spec-Gen" and "Gen-Gen" will become "Spec-Spec" and "Gen-Spec", respectively (Fig. 8C, D). Indirect specialists that establish in motif groups "Spec-Spec" and "Gen-Spec" will not transform the motif group, therefore those cells are also greyed out. Motif group transformations can also result from extinctions and subsequent establishments of species in the opposite guild. For instance, focal generalists can transition to specialists due to extinctions of direct partners and focal specialists can transition to generalists due to the subsequent colonization of direct partners. While this contributes to the frequency of each motif group in the table, we don't calculate the rate of these occurrences because we are only focused on intra-guild indirect effects.

Species guild	Assembly model	Motif group	Motif frequency after establishment	Indirect specialist extinction number | rate	Motif frequency after extinctions	Indirect specialist establishment number | rate	Motif frequency after subsequent establishments
Plant	W/ AF	Spec-Spec	60	58 | 0.97	2		2
		Spec-Gen	1722		1780	54 | 0.03	1007
		Gen-Spec	10	6 | 0.60	4		62
		Gen-Gen	130		136	5 | 0.04	851
	W/O AF	Spec-Spec	187	11 | 0.06	229		193
		Spec-Gen	609		572	37 | 0.06	369
		Gen-Spec	1339	734 | 0.55	655		640
		Gen-Gen	1510		2189	88 | 0.04	2443
Pollinator	W/ AF	Spec-Spec	809	30 | 0.04	1353		1317
		Spec-Gen	1059		705	40 | 0.06	629
		Gen-Spec	2189	17 | 0.01	2732		2796
		Gen-Gen	1034		301	16 | 0.05	349
	W/O AF	Spec-Spec	488	16 | 0.03	567		515
		Spec-Gen	1689		1661	151 | 0.09	1079
		Gen-Spec	1142	274 | 0.24	969		1030
		Gen-Gen	1596		1718	161 | 0.09	2291

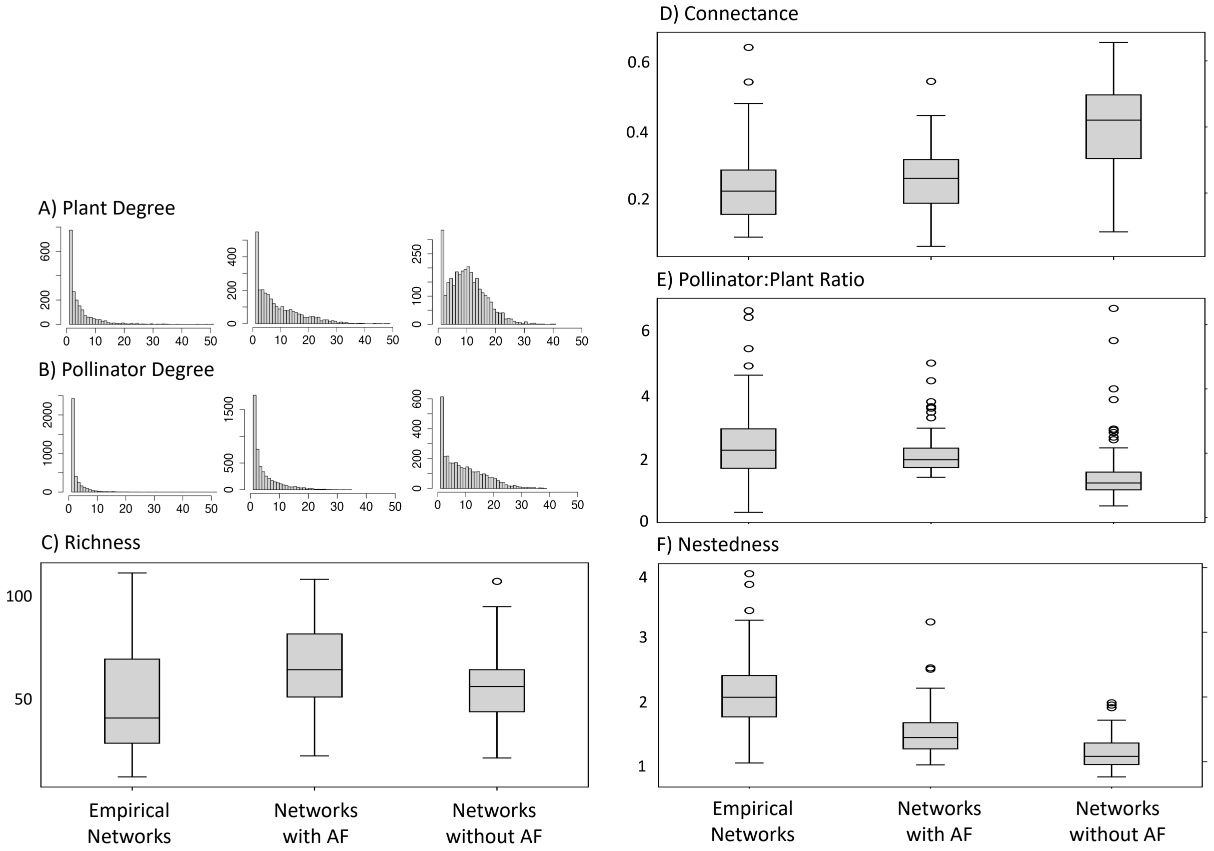

Figure S1: Simulated and empirical network structures and degree distributions.

We performed one-sided Welch t-tests to statistically evaluate the hypotheses that networks assembled from the model with adaptive foraging are richer, less connected, have a higher pollinator:plant ratio, and are more nested than networks assembled from the model without adaptive foraging. The Welch tests were paired to compare networks populated by specialist plants and specialist pollinators at the same probability. We found strong evidence that networks produced by the assembly model with adaptive foraging are richer (C; t-value -5.83; p-value 2.43e-8; df 120) less connected (D; t-value 19.53; p-value 2.23e-39; df 120), have a higher pollinator:plant ratio (E; t-value -6.51; p-value 9.08e-10; df 120), and are more nested (F; t-value -9.71; p-value 7.18e-17; df 120) than networks produced by the assembly model without adaptive foraging.

We performed two-sided Welch t-tests to statistically evaluate the hypotheses that the connectance, plant:pollinator ratio, and richness of empirical networks is significantly different from simulated networks produced from our assembly model with adaptive foraging. Across all structural metrics, networks produced from the assembly model with adaptive foraging were more similar to empirical networks than networks produced from the assembly model without adaptive foraging. However, empirical networks were still significantly less connected (D; t-value -1.97; p-value 0.05; df = 264), had a moderately greater pollinator:plant ratio (E; t-value 2.06; p-value 0.04; df = 264), and were significantly more nested (F; t-value 11.01; p-value 6.54e-23; df = 264) than networks produced from the assembly model with adaptive foraging.

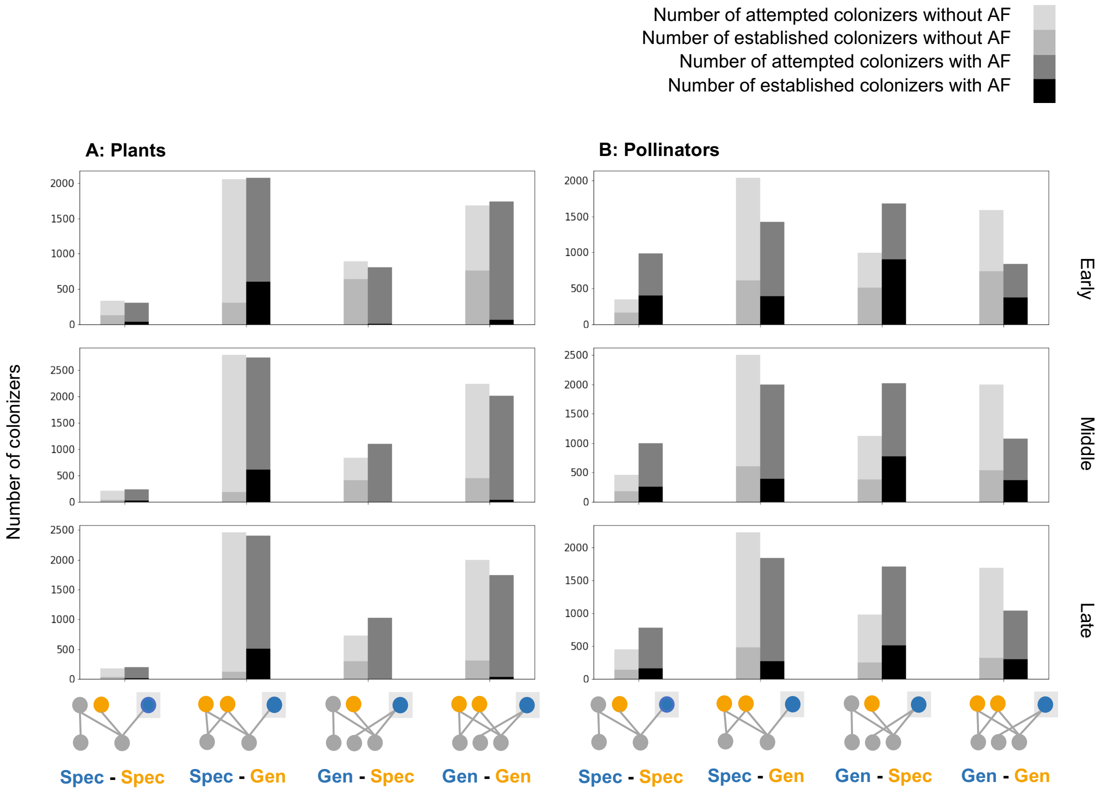

Figure S2: Motif frequency among attempted and established early, middle, and late-stage colonizers for each guild and assembly model.

Here we consider species' motif groups at the moment they arrive to the network before undergoing any transformations due to extinctions or subsequent establishments. The number of attempted (light grey) and established (medium grey) colonizers in the assembly model without adaptive foraging (AF) and the number of attempted (dark grey) and established (black) colonizers in the assembly model with AF are shown for both plants (A) and pollinators (B). These distributions are separated among species that colonize at early stages of assembly (before timestep 33,333), middle stages of assembly (between timesteps 33,333 and 66,666), and late stages of assembly (after timestep 66,666). The motif distribution of attempted colonizers does not vary significantly between early, middle, and late-stage colonizers. However, early colonizers establish at a higher rate than later colonizers.
