# Supplementary material for: The role of intra-guild indirect interactions in assembling plant-pollinator networks
Source: Nat Commun. 2023 Sep 18;14:5797. doi: 10.1038/s41467-023-41508-y (PMC10507117; doi:10.1038/s41467-023-41508-y)
Supplement: Supplementary file 3 — Reporting Summary [file 41467_2023_41508_MOESM3_ESM.pdf]

Corresponding author(s): Sabine Dritz, Fernanda Valdovinos
Last updated by author(s): 8/21/2023

Reporting Summary

Nature Portfolio wishes to improve the reproducibility of the work that we publish. This form provides structure for consistency and transparency in reporting. For further information on Nature Portfolio policies, see our Editorial Policies and the Editorial Policy Checklist.

Statistics

For all statistical analyses, confirm that the following items are present in the figure legend, table legend, main text, or Methods section.

n/a	Confirmed	
☐	☒	The exact sample size (*n*) for each experimental group/condition, given as a discrete number and unit of measurement
☐	☒	A statement on whether measurements were taken from distinct samples or whether the same sample was measured repeatedly
☐	☒	The statistical test(s) used AND whether they are one- or two-sided<br>*Only common tests should be described solely by name; describe more complex techniques in the Methods section.*
☐	☒	A description of all covariates tested
☒	☐	A description of any assumptions or corrections, such as tests of normality and adjustment for multiple comparisons
☒	☐	A full description of the statistical parameters including central tendency (e.g. means) or other basic estimates (e.g. regression coefficient) AND variation (e.g. standard deviation) or associated estimates of uncertainty (e.g. confidence intervals)
☐	☒	For null hypothesis testing, the test statistic (e.g. *F*, *t*, *r*) with confidence intervals, effect sizes, degrees of freedom and *P* value noted<br>*Give P values as exact values whenever suitable.*
☒	☐	For Bayesian analysis, information on the choice of priors and Markov chain Monte Carlo settings
☒	☐	For hierarchical and complex designs, identification of the appropriate level for tests and full reporting of outcomes
☒	☐	Estimates of effect sizes (e.g. Cohen's *d*, Pearson's *r*), indicating how they were calculated

Our web collection on statistics for biologists contains articles on many of the points above.

Software and code

Policy information about availability of computer code

Data collection	Code Availability: Michael Egan and Fernanda Valdovinos developed a MATLAB toolbox called "Plant-Pollinator_Network_Builder.mltbx" to perform simulated assembly of plant-pollinator networks based on Fernanda Valdovinos' theoretical consumer-resource model of plant-pollinator interactions. Sabine Dritz additionally developed code to perform the motif analysis presented in this study. The toolbox, code, and instructions to reproduce the results of this study are available at the project's GitHub repository v1.00 https://github.com/Valdovinos-Lab/Motif_Assembly.
Data analysis	

For manuscripts utilizing custom algorithms or software that are central to the research but not yet described in published literature, software must be made available to editors and reviewers. We strongly encourage code deposition in a community repository (e.g. GitHub). See the Nature Portfolio guidelines for submitting code & software for further information.

Data

Policy information about availability of data

All manuscripts must include a data availability statement. This statement should provide the following information, where applicable:

- Accession codes, unique identifiers, or web links for publicly available datasets
- A description of any restrictions on data availability
- For clinical datasets or third party data, please ensure that the statement adheres to our policy

Data Availability: Simulated plant-pollinator network assembly data can be reproduced with the code and instructions available at the project's GitHub repository v1.0.0: https://github.com/Valdovinos-Lab/Motif_Assembly. Empirical plant-pollinator network data obtained from the Web of Life: ecological networks database is available here: https://www.web-of-life.es/map.php?type=5.

Human research participants

Policy information about studies involving human research participants and Sex and Gender in Research.

Reporting on sex and gender	Our study does not include human research participants.
Population characteristics	Our study does not include human research participants.
Recruitment	Our study does not include human research participants.
Ethics oversight	Our study does not include human research participants.

Note that full information on the approval of the study protocol must also be provided in the manuscript.

Field-specific reporting

Please select the one below that is the best fit for your research. If you are not sure, read the appropriate sections before making your selection.

☐ Life sciences ☐ Behavioural & social sciences ☒ Ecological, evolutionary & environmental sciences

For a reference copy of the document with all sections, see nature.com/documents/nr-reporting-summary-flat.pdf

Ecological, evolutionary & environmental sciences study design

All studies must disclose on these points even when the disclosure is negative.

Study description	To determine how species' indirect interactions and adaptive foraging dynamics influence the assembly of plant-pollinator networks we performed two groups of assembly simulations. One group with adaptive foraging dynamics and the other group without adaptive foraging dynamics. Each group included 121 simulations of network assembly. Each simulation of network assembly was uniquely characterized by a combination of two probabilities: the probability that colonizing plants would we specialists (species only having one interaction) and the probability that colonizing pollinators would be specialists. These probabilities ranged from 0 to 1 by 0.1, 11 values total accounting for 121 assembly simulations within each group. Each simulation ran for 10,000 time steps with 3 plants and 3 pollinators attempting to colonize the network every 2000 time steps. To determine how indirect species interactions influence assembly we pooled data from all successful colonizers in all assembly simulations within a group given those colonizers have indirect interactions in the network. With this data we used motif groups to describe which species were indirectly connected to the colonizer, whether those species went extinct, and whether subsequent colonizers sharing an mutualistic parter with the focal colonizer would establish in the network. To compare resulting network structures with and without adaptive foraging we calculated the connectance, richness, and nestedness of the 121 assembled networks in each group. We then used one sided paired Welch's t tests to determine whether each structural metric varied significantly between the groups. Lastly we used a two sided Welch's t test to compare network structures assembled with adaptive foraging to observed network structures using 117 empirical networks from the Web of Life: ecological networks database. This database includes 156 networks but we excluded the 11 that contained disconnected modules as a result of being very small and 28 networks that were larger than 110 species (the maximum network size produced by our assembly models) to more accurately compare other structural statistics.
Research sample	Because our simulations do not include empirical data, the mechanisms are generalizable within the model's assumptions. The model assumes that mutualisms between plants and pollinators are obligate (meaning they do not experience population growth in the absence of the mutualism). The model also assumes that pollinators are aware of all resources in the field and can quickly respond to variation in the resource landscape through their foraging behavior. Lastly, the model assumes that plant abundance is more strongly determined by inter and intraspecific competition than pollination services. All of these assumptions are supported by empirical data but do not apply to all pollination networks.
Sampling strategy	We chose to vary simulations based on the probability of colonizers being specialists because specialists have unique relationships with their direct and intirect interaction partners (by providing exclusive mutualistic resources in the case of plants or high quality pollination services in the case of pollinators, but also being most sensitive to disturbance) and because they contribute uniquely to

	network structure (by having only one interaction).
Data collection	The empirical data was sourced from Web of Life: ecological network database which includes empirical networks from around the world.
Timing and spatial scale	Our simulations are meant to represent the assembly of plant-pollinator networks over ecological, rather than evolutionary, timescales as a result of species invasions, extinctions, and range shifts. Given the doubling time of pollinator populations our assembly simulations roughly span 144 years and species colonizations and extinctions occur every 3 years.
Data exclusions	We excluded colonizers who do not have any indirect interactions, and therefore form distinct modules, from our assembly simulations because they do not address our primary research question. Distinct modules occurred in our simulations rarely and always became reconnected to the network during subsequent colonization events. We additionally excluded the first 3 colonizers of each guild because the network was too small to analyze.
Reproducibility	The code for our assembly model as well as all parameter values used will be published on GitHub so that it is reproducible. To compute the nestedness of assembled networks we used the maxnodf R package. The motif groups we developed for this project are clearly defined and could be identified in other bipartite networks.
Randomization	All model parameters were held constant across all simulations in both groups except for G which controls adaptive foraging dynamics (equal to 1 in the group with adaptive foraging and 0 in the group without adaptive foraging). Parameter values are sampled from uniform distributions (the mean and variance of each distribution is included in the SI). To ensure that parameters were the same across all simulations we set a random seed.
Blinding	This is not relevant to our study because all data was simulated.

Did the study involve field work? ☐ Yes ☒ No

Reporting for specific materials, systems and methods

We require information from authors about some types of materials, experimental systems and methods used in many studies. Here, indicate whether each material, system or method listed is relevant to your study. If you are not sure if a list item applies to your research, read the appropriate section before selecting a response.

Materials & experimental systems

n/a	Involved in the study
☒	☐ Antibodies
☒	☐ Eukaryotic cell lines
☒	☐ Palaeontology and archaeology
☒	☐ Animals and other organisms
☒	☐ Clinical data
☒	☐ Dual use research of concern

Methods

n/a	Involved in the study
☒	☐ ChIP-seq
☒	☐ Flow cytometry
☒	☐ MRI-based neuroimaging

March 2021
